# Metabolically Controlled Oncological Treatment: An Evidence-Graded Framework Integrating Metabolic Reprogramming, Targeted Drug Delivery, and Standard Oncological Care

**DOI:** 10.3390/molecules31213970

**Published:** 2026-11-03

**Authors:** Mehmet Salih Iyikesici, Oral Oncul, Sena Sen, Sirin Budak, Thomas N. Seyfried, Tomas Duraj

**Affiliations:** 1 Neolife Oncology Centre, 34340 Istanbul, Türkiye; drsalihiyikesici@gmail.com (M.S.I.); sirin_budak@hotmail.com (S.B.); 2 Department of Infectious Diseases and Clinical Microbiology, Istanbul Faculty of Medicine, Istanbul University, 34093 Istanbul, Türkiye; 3 Department of Basic Oncology, Oncology Institute, Istanbul University, 34093 Istanbul, Türkiye; sena.sen@istanbul.edu.tr; 4 Department of Biology, Boston College, Boston, MA 02467, USA; thomas.seyfried@bc.edu (T.N.S.); tomas.duraj@bc.edu (T.D.)

**Keywords:** cancer metabolism, metabolic reprogramming, tumor microenvironment, targeted drug delivery, evidence-based oncology

## Abstract

Cancer cells exhibit metabolic reprogramming, including increased aerobic glycolysis, glutaminolysis, and altered lipid metabolism, which supports tumor growth and contributes to immune evasion. A growing but heterogeneous body of literature has investigated whether targeting these metabolic dependencies alongside standard oncologic treatment may provide therapeutic benefit. This review critically examines clinically investigated metabolically directed approaches, including metronomic chemotherapy, hyperthermia, high-dose intravenous vitamin C, hyperbaric oxygen therapy, fasting-related nutritional strategies, ketogenic diets, and insulin-based approaches, while distinguishing them from established targeted therapies, monoclonal antibodies, and immune checkpoint inhibitors. Emerging immunometabolic and microenvironmental targets are also discussed, including lactate transport, ferroptosis, autophagy and amino acid metabolism, cancer-associated fibroblasts, tumor-associated macrophages, abnormal vasculature, and immunosuppressive cytokine signaling. In addition, drug-delivery strategies are considered by distinguishing the established pharmaceutical role of dimethyl sulfoxide (DMSO) as a solvent or excipient from nanoparticle-, exosome-, and peptide-based targeting platforms. Available evidence is characterized according to clinical maturity, ranging from established phase III-supported therapies and randomized clinical evidence to early-phase, preclinical, or currently unproven approaches. Although several metabolically directed interventions have biological rationale and varying degrees of clinical support, no comprehensive multicomponent metabolic regimen has been validated in controlled clinical trials. Future development should prioritize stepwise, biomarker-informed prospective studies evaluating individual interventions and biologically justified combinations alongside standard-of-care therapy. Metabolically directed strategies should therefore be interpreted according to the evidence supporting each component rather than as a single established therapeutic regimen.

## 1. Introduction

Cancer is the second most common cause of death worldwide, and surgery, chemotherapy, and radiotherapy remain the fundamental approaches to its treatment [1]. The limited cellular specificity of conventional chemotherapeutic agents leads to systemic toxicity, while tumor heterogeneity (spanning genomic instability, clonal evolution, cancer-stem-cell plasticity, and dynamic remodeling of the tumor microenvironment) paves the way for the development of resistance and relapse over time [2]. Over the past two decades, immunotherapy, including checkpoint blockade, adoptive T-cell transfer, and cancer vaccines, has become a major axis of cancer treatment; however, these approaches can also carry serious immune-related adverse effects [3].

Against this background, this review focuses on metronomic chemotherapy, hyperthermia, high-dose vitamin C, hyperbaric oxygen therapy (HBOT), insulin-based approaches, and dimethyl sulfoxide (DMSO), selected because of their proposed effects on tumor metabolism, oxygenation, oxidative stress, drug sensitivity, and/or the tumor microenvironment. These interventions are considered in the context of metabolic reprogramming and alongside established targeted therapies, monoclonal antibodies, and immunotherapies. The review is not intended as an exhaustive survey of complementary or alternative cancer therapies, nor does inclusion imply equivalent efficacy or evidentiary support. Instead, it distinguishes established oncological strategies from interventions that remain investigational or lack adequate clinical validation, with the aim of separating biologically plausible concepts that warrant prospective investigation from therapeutic claims not yet supported by robust clinical evidence.

This review critically evaluates metabolically directed therapeutic approaches based on the maturity and quality of the available evidence. The appraisal considers five principal criteria: (i) study design and stage of clinical development (randomized controlled trials, prospective clinical studies, retrospective analyses, case series, and preclinical investigations); (ii) reported clinical outcomes (overall survival, progression-free survival, objective tumor response, treatment-related toxicity, and quality of life); (iii) consistency and reproducibility of findings across independent studies; (iv) methodological limitations; and (v) the current clinical and regulatory status of each intervention. Evidence categories are used descriptively to distinguish established, clinically supported therapies from investigational approaches supported by preliminary clinical observations or preclinical findings.

## 2. Biological Rationale for Metabolically Directed Oncology

**Figure 1:**
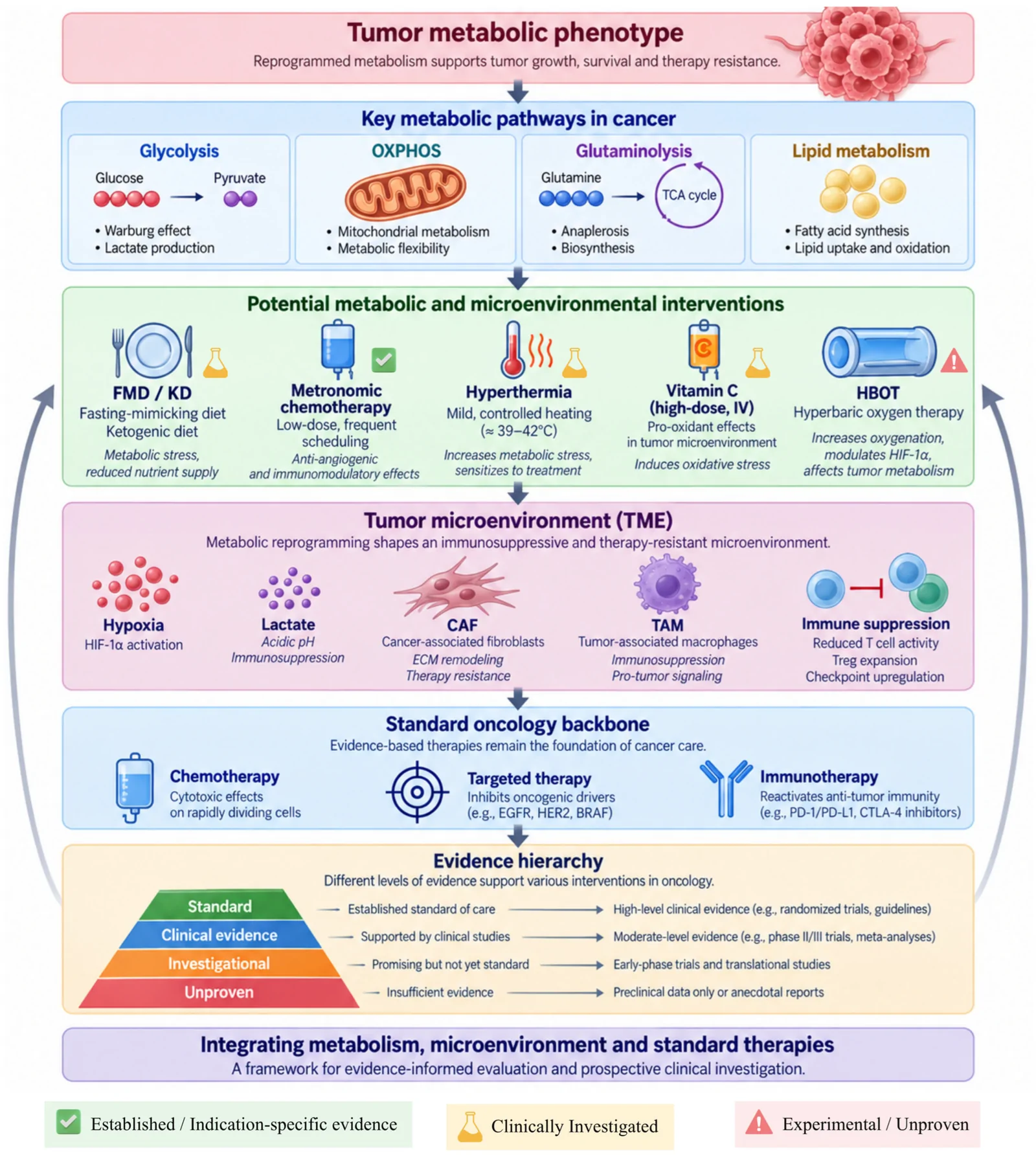
Conceptual framework of metabolically directed oncology and its integration with the tumor microenvironment and standard oncological care. Arrows represent conceptual relationships and do not imply established therapeutic synergy, treatment sequence, or clinical efficacy. This figure was created using Canva and Microsoft PowerPoint. Evidence categories are intervention- and indication-specific. Inclusion of an intervention in the conceptual framework does not imply established clinical efficacy or endorsement of combined use. The arrows indicate the conceptual relationships and interactions among tumor metabolic pathways, metabolic interventions, the tumor microenvironment, and standard oncological therapies. The background colors distinguish the major components of the proposed framework, while the color-coded symbols indicate the evidence status of the interventions: green (established or indication-specific evidence), yellow (clinically investigated), and red (experimental or unproven). The colors do not imply equivalent clinical efficacy across interventions.

The conceptual framework of this review is summarized in Figure 1. Tumor metabolic reprogramming is considered in relation to metabolically directed interventions, the tumor microenvironment, and the standard oncological treatment backbone, while recognizing that these approaches are supported by substantially different levels of clinical evidence. Evidence strength was categorized qualitatively as strong, moderate, limited, or unproven, based primarily on study design, demonstrated clinical benefit, consistency of findings, and methodological quality. Strong evidence generally reflects well-established clinical benefit supported by randomized trials, whereas moderate evidence indicates promising but incompletely validated clinical findings. Limited evidence refers to early-phase, uncontrolled, or observational studies, while unproven approaches lack adequate clinical evidence of anticancer efficacy. These categories represent a descriptive assessment rather than a formal GRADE evaluation.

### 2.1. The Warburg Effect and Metabolic Flexibility

The observation described by Otto Warburg in the 1920s—that cancer cells shift strongly toward aerobic glycolysis even in the presence of oxygen (the Warburg effect)—forms the foundation of modern cancer metabolism research [4]. Today, this concept of “metabolic flexibility” is regarded as a multidimensional network encompassing glycolysis, glutaminolysis, fatty-acid oxidation and synthesis (FASN-mediated de novo lipogenesis), the mTOR signaling pathway, and one-carbon/antifolate metabolism [5]. Simultaneous suppression of several of these pathways to drive the tumor cell into an energy and biosynthetic-precursor crisis provides the scientific rationale for the concept of “metabolic blockade” [6,7,8,9]. Beyond glycolysis and glutaminolysis, lipid metabolic reprogramming represents another important mechanism by which cancer cells adapt to metabolic stress. Increased fatty acid oxidation (FAO), particularly through CPT1-dependent pathways, is traditionally hypothesized to support energy production and cell survival under nutrient deprivation or therapeutic stress [10]. However, recent evidence also suggests that lipid droplet accumulation and lipid-related metabolic rewiring are indicative of oxidative phosphorylation insufficiency, with enhanced fatty acid synthesis and uptake but limited β-oxidation [11,12]. Similarly, enhanced de novo lipogenesis, regulated by fatty acid synthase (FASN) and SREBP1, contributes to membrane biosynthesis and tumor growth, while alterations in cholesterol metabolism, including changes in the mevalonate pathway and SREBP2-mediated regulation, influence membrane organization and oncogenic signaling [13]. These metabolic adaptations may promote resistance to chemotherapy and targeted therapies. In addition, changes in cellular lipid composition can modulate susceptibility to lipid peroxidation and ferroptosis. Although targeting these pathways is a promising therapeutic strategy, their clinical utility remains investigational and requires further validation [14].

Agents targeting mitochondrial respiratory function, including metformin, IACS-010759, and atovaquone, have been investigated in cancer research. However, inhibition of oxidative phosphorylation (OXPHOS) may induce compensatory metabolic responses, particularly increased glycolytic activity, as demonstrated in experimental models of respiratory inhibition and atovaquone-mediated complex III blockade [15,16,17]. These adaptive responses highlight the limitations of targeting a single metabolic pathway and provide a rationale for investigating carefully selected multipathway strategies [7,18,19]. Nevertheless, the clinical development of potent OXPHOS inhibitors, including IACS-010759, has been constrained by treatment-related toxicity [18,19]. Therefore, future metabolic interventions should consider tumor-specific metabolic dependencies, compensatory mechanisms, and potential effects on normal tissues [20,21].

### 2.2. Metabolic Phenotyping/Seahorse

A central methodological question in metabolically directed oncology is how to measure, rather than assume, the glycolytic and oxidative phenotype of a tumor. Extracellular flux analysis, commercially available through the Agilent Seahorse XF/XFe platform, measures the oxygen consumption rate (OCR) as an indirect readout of mitochondrial oxidative phosphorylation and the extracellular acidification rate (ECAR) as a surrogate for glycolytic activity in living cells [22,23]. Combined OCR/ECAR profiling can therefore characterize metabolic phenotypes and assess changes in cellular bioenergetics in response to experimental interventions [24,25].

Extracellular flux analysis is well established in cancer cell lines and is increasingly used in patient-derived organoids and short-term ex vivo models [26,27]. However, its application for individualized treatment selection remains investigational. In addition, extracellular flux measurements are highly sensitive to culture conditions, nutrient availability, and ex vivo handling. Placement of cells into culture can itself induce rapid metabolic alterations, including the Crabtree effect, and differences in media composition or experimental conditions may substantially influence the apparent glycolytic or oxidative phenotype [25,28]. Importantly, in vitro OCR/ECAR measurements may not fully reflect the complexity of the in vivo tumor microenvironment, which is characterized by spatial heterogeneity, limited nutrient availability, hypoxia, and dynamic interactions among cancer, stromal, and immune cells. Indeed, the same cell lines may display markedly different metabolic profiles under different culture and laboratory conditions [29]. Tumor metabolism is also dynamic and may adapt under therapeutic pressure; therefore, a single baseline OCR/ECAR measurement characterizes the metabolic state at a particular time point rather than predicting the subsequent adaptive trajectory of the tumor [2,16,17]. Accordingly, Seahorse-based profiling should currently be viewed as a research tool for metabolic characterization and hypothesis generation, and as a potential correlative biomarker in prospective studies, rather than as a validated clinical decision-making platform [30].

## 3. Clinically Investigated Metabolic Interventions

### 3.1. Metronomic Chemotherapy

Metronomic chemotherapy (MCT), in contrast to the standard maximum-tolerated-dose (MTD) regimen, involves administering cytotoxic agents at low doses, at frequent intervals, without long drug-free breaks [31]. Its primary mechanism of action is described as anti-angiogenesis, achieved by targeting the endothelial cells that supply tumor vasculature and circulating endothelial progenitor cells; immune modulation via reduction of regulatory T cells and promotion of dendritic-cell maturation has also been demonstrated [31,32]. A systematic review of controlled phase II/III trials published between 2012 and 2018 found positive results in four phase III and four randomized phase II studies; however, most studies did not demonstrate statistically significant improvements in overall survival or progression-free survival [33]. These findings highlight the heterogeneity of clinical outcomes across tumor types, treatment settings, and evaluated endpoints, limiting the generalizability of the reported benefits. Although MCT may offer potential advantages in selected clinical settings, particularly for frail or elderly patients and in palliative care, its clinical utility depends on tumor type, treatment intent, and the specific regimen used. Current evidence does not support its general substitution for standard maximum-tolerated-dose chemotherapy in curative settings [31,33,34]. Combining MCT with other multimodal therapeutic strategies, such as metabolic inhibitors, pro-oxidant therapies or repurposed anti-cancer drugs, remains under investigation.

### 3.2. Hyperthermia (Regional or Systemic Heat Therapy)

Hyperthermia involves the controlled application of heat to enhance tumor sensitivity to conventional treatments through mechanisms including altered blood flow, protein damage, and modulation of immune responses [35,36,37,38]. Regional hyperthermia, which selectively heats tumor-bearing tissues, has been investigated as an adjunct to radiotherapy and chemotherapy. Randomized clinical trials have demonstrated improvements in tumor response and local control, particularly in locally advanced cervical cancer, although findings in other tumor types, including rectal and bladder cancers, have been less consistent [39]. Systemic or whole-body hyperthermia, in contrast, exposes the entire body to elevated temperatures and may induce broader physiological and immunological effects. However, its clinical evidence remains limited, and a consistent survival benefit has not been established in randomized controlled trials [40]. Heat-induced expression of heat-shock proteins, particularly HSP70, may contribute to immune modulation through natural killer cells and dendritic cells [41,42,43]. Thus, the clinical benefits observed with regional hyperthermia should not be extrapolated to systemic hyperthermia, and both approaches require indication-specific evaluation.

### 3.3. High-Dose Intravenous Vitamin C (Pro-Oxidant Effect)

At physiological concentrations achieved through oral intake, ascorbic acid (vitamin C) primarily acts as an antioxidant [44]. In contrast, pharmacological concentrations reached through intravenous administration may exert pro-oxidant effects, including extracellular generation of hydrogen peroxide (H_2_O_2_). Reduced catalase activity in certain cancer cells has been proposed to limit their capacity to detoxify peroxide and thereby contribute to the potential preferential cytotoxic effects of pharmacological ascorbate on tumor cells [44,45,46]. However, tumor sensitivity to pharmacological ascorbate may vary according to intrinsic antioxidant capacity and metabolic characteristics. In addition to catalase activity, glutathione-dependent antioxidant systems, glutathione peroxidases, and peroxiredoxins contribute to cellular hydrogen peroxide detoxification and may influence susceptibility to oxidative damage [47,48]. Differences in mitochondrial function, iron metabolism, and intracellular redox balance may further modify tumor responses to pharmacological ascorbate. Importantly, these mechanisms have been demonstrated predominantly in preclinical models, whereas their clinical relevance and predictive value in patients remain insufficiently established [49]. Therefore, the proposed selective pro-oxidant effects should not be interpreted as evidence of consistent antitumor efficacy across different cancer types.

Phase I–II studies suggest that high-dose intravenous vitamin C is feasible and generally well tolerated when administered alongside chemotherapy in selected patient populations, with some studies also reporting potential quality-of-life benefits [46,50,51]. Nevertheless, robust phase III evidence demonstrating improvements in clinically relevant outcomes, particularly overall survival, remains insufficient [45,46,52]. Accordingly, high-dose intravenous vitamin C should currently be regarded as an investigational supportive or adjunctive approach whose clinical efficacy has not yet been established conclusively. Similarly, the proposed enhancement of antitumor activity when pharmacologic ascorbate is combined with hyperbaric oxygen or other strategies targeting the tumor microenvironment is based largely on mechanistic and preclinical rationale and requires confirmation in prospective human studies [46,53,54,55]. From a safety perspective, the potential risk of hemolysis associated with high-dose intravenous vitamin C in individuals with glucose-6-phosphate dehydrogenase (G6PD) deficiency should be considered, and assessment of G6PD status before treatment may be appropriate [56,57]. Baseline renal function should be assessed using serum creatinine and estimated glomerular filtration rate (eGFR) before initiating high-dose intravenous vitamin C, with subsequent monitoring when clinically indicated because of the potential risk of oxalate nephropathy.

### 3.4. Hyperbaric Oxygen Therapy

Hyperbaric Oxygen Therapy (HBOT) involves keeping the patient in a pressurized environment with 100% oxygen. Tumor hypoxia is known to trigger HIF-1α–mediated signaling pathways (angiogenesis, glycolytic shift, apoptosis resistance) [58]; it has therefore been suggested that reducing hypoxia with HBOT could theoretically work against the tumor through both reoxygenation and increased mitochondrial ROS production (pro-oxidative effects) [59,60]. In some preclinical models, HBOT has been shown to suppress HIF-1α, slowing tumor growth and increasing apoptosis [61,62]. HBOT has been reported to suppress tumor growth when given in a combination regimen with the ketogenic diet [63,64], but also mildly increase both proliferation and paradoxically chemosensitivity via the HIF1α/HIF2α–ABCG2 axis when administered as monotherapy [65]. Current reviews conclude that there is, overall, no evidence that HBOT promotes tumor growth as a general rule, while emphasizing that the effect varies by tumor type, stage, and concomitant treatment [62,65]. However, to our knowledge, no clinical report of accelerated tumor progression or hyperprogressive disease with HBOT has been presented, despite increasing clinical use and FDA-approved indications such as radiation necrosis [66].

Accordingly, the stand-alone anticancer role of HBOT remains investigational. Although combining HBOT with hyperthermia, high-dose vitamin C, or chemotherapy has a mechanistic rationale, robust confirmatory Phase III evidence demonstrating improved survival or consistent clinical benefit remains insufficient. Furthermore, the available findings are limited by heterogeneous treatment protocols, tumor-specific responses, and difficulties in distinguishing the effects of HBOT from those of concomitant therapies. These combinations should therefore be regarded as hypotheses requiring prospective clinical evaluation rather than established treatment strategies.

### 3.5. Nutrition-Based Metabolic Interventions: Fasting-Mimicking Diet and the Ketogenic Diet

Fasting-related nutritional strategies have emerged as clinically investigated approaches to modulating cancer metabolism. In the multicenter randomized phase II DIRECT trial, patients with HER2-negative breast cancer who followed a fasting-mimicking diet (FMD) before and during neoadjuvant chemotherapy showed a higher radiological complete/partial response rate, together with reduced chemotherapy-induced DNA damage in normal cells [67]. A separate randomized study reported lower rates of grade III/IV nausea and neutropenia in patients receiving an FMD during chemotherapy [68]. These findings provide clinical support for further investigation of structured, medically supervised FMD as an adjunct to standard cancer therapy, although confirmation in larger trials and across different tumor types is required. Importantly, improvements in chemotherapy tolerability should not be interpreted as evidence of enhanced antitumor efficacy or improved survival. Although some studies have reported favorable radiological responses, the effects of FMD on progression-free and overall survival remain uncertain. Furthermore, dietary restriction may exacerbate malnutrition, cancer-associated weight loss, and loss of lean body mass, particularly in nutritionally vulnerable patients. Therefore, careful patient selection, nutritional assessment, and medical supervision are essential when investigating these interventions [69].

While there is ample preclinical evidence for the anti-cancer benefits of the ketogenic diet (KD) [70], the clinical evidence is still emerging. Preclinical studies have suggested that KDs may enhance the antitumor activity of PI3K inhibition in pancreatic-cancer models [71,72], as well as monotherapy and synergy with other treatments [73]; however, these findings cannot yet be extrapolated to broad clinical efficacy in patients with cancer.

Pilot clinical studies have investigated KDs primarily in glioblastoma [20,74,75], with larger phase II studies underway (NCT05708352). Although maintaining a low glucose-ketone index (GKI) has been proposed as a potential metabolic biomarker of compliance [20,74,76], its association with improved clinical outcomes remains to be fully established across larger patient cohorts. The clinical testing of KDs and by extension nutritional ketosis is also complicated by the need for sustained long-term adherence, frequent biomarker testing (GKI, ideally via continuous glucose/ketone monitoring), and a lack of funding incentives, with most clinical trials being investigator-initiated pilot studies [20]. At this time, neither FMD nor KDs should be considered a substitute for guideline-based oncological treatment, but provide an opportunity to leverage systemic diet-induced metabolic changes to potentially synergize with standard treatments.

### 3.6. Insulin-Augmented Low-Dose Chemotherapy

Two related but distinct approaches exist in the literature and should not be conflated. “Insulin Potentiation Therapy” (IPT) refers to a hypothesis originating in the 1930s, most recently presented in [77,78], holding that insulin-induced increases in cell-membrane glucose-transporter activity allow very low doses of chemotherapy (10–25% of standard dose) to achieve efficacy equivalent to full-dose treatment. Available clinical evidence does not establish that insulin potentiation therapy can achieve standard-dose chemotherapy efficacy using substantially reduced drug doses, and the approach may pose risks, including hypoglycemia [77,78]. Based on current evidence, IPT should not be used as a substitute for standard-dose chemotherapy, and no claim that it increases drug uptake or potentiates efficacy should be made outside a controlled trial.

A separate line of published clinical research, generally termed “metabolically supported chemotherapy”, has been reported by İyikesici, Seyfried, and colleagues: full- or near-full-dose standard chemotherapy regimens administered concomitantly with pharmacologically induced, closely monitored hypoglycemia (via regular insulin), together with a ketogenic diet, hyperthermia, and hyperbaric oxygen therapy [79]. The proposed rationale involves insulin-mediated metabolic modulation and potential enhancement of tumor-cell sensitivity to chemotherapy. However, increased glucose uptake does not necessarily correspond to increased intracellular accumulation of chemotherapeutic agents. Direct experimental evidence demonstrating that insulin-induced hypoglycemia enhances tumor-selective drug uptake remains insufficient, and this mechanism has not been clinically validated. Published outcomes for this specific protocol consist of case reports and small retrospective case series in stage III/IV breast, gastric, pancreatic, and non-small-cell lung cancer, reporting favorable radiological or pathological responses and, in some series, extended survival compared with historical controls [34,79,80,81,82,83]. These reports are hypothesis-generating and mechanistically distinct from historical low-dose IPT in that standard-dose chemotherapy is retained. However, these findings should be interpreted cautiously due to several methodological limitations. Retrospective study designs introduce potential selection bias, while comparisons with historical controls may be confounded by differences in patient characteristics, disease severity, and treatment practices. Furthermore, the simultaneous administration of chemotherapy, induced hypoglycemia, ketogenic diet, hyperthermia, and HBOT prevents attribution of the observed outcomes to any individual component. Consequently, these studies cannot establish a causal relationship between the metabolic interventions and the reported clinical benefits.

Moreover, these studies were conducted by a single treating group without independent replication. Because the induction of hypoglycemia is an active medical intervention with recognized risks, its safe administration requires intensive glucose monitoring and appropriate medical supervision. This protocol separately from historical low-dose IPT; however, neither approach has sufficient controlled clinical evidence to support its recommendation as standard oncological treatment.

## 4. Targeted Drug Delivery Strategies and the Role of DMSO

Improving tumor-selective drug delivery is an important objective in oncology, particularly when systemic toxicity restricts the therapeutic window of cytotoxic or targeted agents [84]. Within this context, DMSO has occasionally been proposed as a permeability-enhancing carrier; however, this concept should be distinguished from engineered drug-delivery platforms specifically designed to improve tumor targeting. This section therefore evaluates the evidence for DMSO as a pharmaceutical carrier and contrasts it with nanoparticle-, exosome-, and peptide-based delivery strategies. In the context of metabolic cancer therapy, targeted delivery platforms may enable selective administration of agents that interfere with glycolysis, glutamine metabolism, lipid metabolism, or tumor-associated metabolic signaling. By improving drug localization and potentially reducing systemic exposure, these strategies could help address the limited tumor specificity and toxicity of certain metabolic inhibitors. However, their ability to enhance metabolic targeting and clinical outcomes remains largely investigational.

### 4.1. DMSO as a Pharmaceutical Solvent and Permeability Enhancer

Dimethyl sulfoxide (DMSO) is a solvent widely used in pharmacology and as a cell membrane permeability enhancer. It has an approved intravesical indication for interstitial cystitis and is also used as a vehicle or excipient in some pharmaceutical formulations [85]. However, there is no clinical evidence that DMSO itself has therapeutic anticancer activity in humans.

The biological effects of DMSO are concentration-dependent. At high concentrations, laboratory studies have demonstrated toxic effects on erythrocytes, platelets, and vascular endothelial cells, including hemolysis, platelet dysfunction, and endothelial apoptosis [86]. At lower concentrations, DMSO has been reported to alter mitochondrial respiratory capacity in cancer cells, with very low concentrations producing variable and, in some experimental settings, potentially proliferation-enhancing effects [87]. Limited clinical observations have also suggested a possible role in the palliation of refractory cancer-related pain, although this is distinct from an anticancer effect.

Accordingly, in this context, the established relevance of DMSO is primarily as a pharmaceutical solvent or permeability-enhancing excipient. Evidence is insufficient to support its use as an anticancer agent or as a tumor-selective chemotherapy carrier, and claims that DMSO potentiates the antitumor efficacy of co-administered chemotherapy have not been confirmed in clinical studies. Unlike engineered tumor-targeted delivery systems, DMSO has not been shown to selectively deliver metabolic inhibitors to cancer cells or improve the clinical efficacy of metabolically directed therapies.

### 4.2. Nanoparticle-Based Drug Delivery

Engineered nanoparticles offer a more extensively investigated approach to tumor-directed drug delivery. Liposomal, polymeric, and inorganic nanoparticles can modify drug pharmacokinetics and biodistribution and may enhance tumor accumulation through passive and/or ligand-mediated targeting mechanisms [88]. Several nanoparticle-based formulations, including PEGylated liposomal doxorubicin, have gained regulatory approval, while ongoing preclinical and clinical research continues to optimize drug loading, stability, tumor penetration, and controlled release [89,90]. These platforms therefore provide clinical proof of principle that reformulating anticancer agents can modify their delivery and toxicity profiles [91]. Nevertheless, the performance of individual nanoparticle systems is formulation- and tumor-dependent, and successful delivery should not be generalized across all nanoparticle technologies. In metabolically directed oncology, nanoparticle platforms are being investigated for the delivery of agents targeting glycolytic enzymes, glutamine metabolism, and other metabolic dependencies. Such systems may improve the localization and controlled release of metabolic inhibitors; however, evidence of improved clinical outcomes specifically attributable to metabolic targeting remains limited [92].

### 4.3. Exosome- and Peptide-Based Targeting

Exosomes are endogenous extracellular vesicles increasingly investigated as delivery vehicles for chemotherapeutic agents, nucleic acids, and proteins. Their biological origin offers potential advantages in biocompatibility and cellular communication, and their surfaces can be engineered to enhance tissue and tumor targeting. However, challenges in isolation, standardization, cargo-loading efficiency, reproducibility, and large-scale manufacturing continue to limit clinical translation [89,93,94].

Tumor-targeting peptides represent another strategy to improve drug localization and tissue penetration. For example, the iRGD cyclic peptide interacts with αv integrins and neuropilin receptors associated with tumor vasculature and has been studied as a conjugated or co-administered approach to enhance tumor penetration of therapeutic agents [95,96]. Although peptide-directed approaches have advanced toward clinical evaluation, their therapeutic utility remains dependent on the specific carrier, payload, molecular target, and tumor context.

Overall, nanoparticle-, exosome-, and peptide-based systems provide a more advanced scientific framework for tumor-directed drug delivery than the proposed use of DMSO as a nonspecific chemotherapy carrier [97]. However, their clinical maturity differs: some nanoparticle formulations are already clinically established, whereas exosome- and peptide-based strategies for metabolic targeting remain largely investigational. Exosome-based platforms may enable the delivery of nucleic acids targeting metabolic enzymes or regulatory pathways, while tumor-targeting peptides could facilitate the selective localization of metabolic inhibitors [98].

## 5. Integration with Standard Precision Oncology

Unlike the metabolic approaches above, the following components are standard oncological treatments backed by strong phase III evidence and included in international guidelines (NCCN, ESMO). Whether combining these treatments with metabolic agents produces synergy in patients has not yet been demonstrated [99]; the discussion below therefore presents mechanistic hypotheses that would need to be tested prospectively in clinical settings, not established combination regimens.

### 5.1. Molecular Targets

Molecularly targeted therapies have become an established component of precision oncology, with treatment selection increasingly guided by actionable genomic alterations. EGFR-mutated tumors, particularly lung cancers, are treated with third- and first-generation TKIs such as osimertinib and erlotinib as standard treatment [100]. In tumors with ALK/ROS1 fusions, agents such as alectinib and crizotinib are standard treatment [101,102]. In BRCA1/2-mutated tumors with impaired DNA repair mechanisms, PARP inhibitors such as olaparib act via the principle of synthetic lethality and are approved in the adjuvant and metastatic settings [103]. In tumors harboring the BRAFV600E mutation, the combination of dabrafenib plus trametinib is standard treatment, depending on tumor type and clinical indication [104].

A mechanistic hypothesis of synergy can be proposed between the oxidative DNA damage produced by high-dose IV vitamin C and the repair-blocking effect of PARP inhibitors [105]. Preliminary clinical observations have been reported in a small case series of eight patients with homologous recombination repair deficiencies receiving this combination, with favorable tumor responses observed [105]. However, these uncontrolled findings cannot establish the efficacy of the combination or determine the contribution of intravenous vitamin C beyond PARP inhibition. Prospective controlled clinical trials are therefore required to evaluate its safety and therapeutic benefit. Likewise, any expectation that hyperthermia, HBOT, or DMSO would meaningfully potentiate PARP-inhibitor activity is speculative and unsupported by clinical data at this time.

### 5.2. By Pathological Features: Monoclonal Antibodies

In HER2-positive breast and gastric cancers, HER2-directed monoclonal antibodies such as trastuzumab and pertuzumab have established roles in treatment [106]. Bevacizumab is also used in selected malignancies in which VEGF-mediated angiogenesis is a relevant therapeutic target [107]. The combination of bevacizumab-based chemotherapy with regional hyperthermia has been examined in small pilot studies [108]. Although these preliminary findings provide a rationale for further investigation of potential interactions between hyperthermia and anti-angiogenic therapy, the available clinical evidence remains limited. Larger, controlled prospective studies are therefore required before any synergistic effect can be confirmed.

### 5.3. By Immunological Features: Checkpoint Inhibitors

PD-1/PD-L1 blockers (pembrolizumab, nivolumab, atezolizumab) are standard treatment in patients selected according to PD-L1 level, MSI status, and TMB values [109]. There is strong mechanistic evidence that lactic-acid accumulation in the tumor microenvironment suppresses T-cell function; for this reason, reversing tumor acidification by metabolic targeting [110] buffering the acidic microenvironment and combining hyperthermia with immunotherapy is an active area of current immune-metabolism research [43,111,112,113]. Whether adding HBOT, high-dose IV vitamin C, insulin-augmented chemotherapy, or DMSO to checkpoint-inhibitor therapy improves outcomes has not been studied and should not be presented as an established strategy; it remains a hypothesis worth testing in the context of the acidic-microenvironment/hyperthermia rationale described above.

## 6. Emerging Metabolic and Microenvironmental Targets

The following subsections summarize current literature that could strengthen the metabolic–immunological rationale for future protocol design. These are largely at the preclinical/early-clinical stage and should be regarded as research hypotheses, not as components of a validated treatment regimen.

### 6.1. Lactate/MCT Axis

Glycolytic tumor cells export lactate predominantly through MCT4, contributing to lactate accumulation and acidification of the tumor microenvironment [113,114,115]. Although closely interconnected, lactate-mediated signaling and extracellular acidification exert distinct biological effects. Reduced extracellular pH can impair dendritic-cell maturation, natural killer cell cytotoxicity, and CD8+ T-cell activation and effector functions. In contrast, lactate can act as a metabolic substrate and signaling molecule, influencing macrophage polarization through mechanisms such as histone lactylation, which may promote immunosuppressive M2-like phenotypes and increased arginase-1 expression. These effects are context dependent and should not be attributed solely to extracellular acidification [112,113]. AZD3965, a selective MCT1 inhibitor, has demonstrated enhanced antiproliferative effects in combination with metformin in preclinical models. However, MCT4 expression may contribute to resistance to MCT1 inhibition [32,115]. Together, these findings support the investigation of lactate transport and extracellular pH regulation as related but distinct therapeutic targets. Although buffering the acidic tumor microenvironment has been proposed as an adjunct to immunotherapy, the clinical benefit of bicarbonate buffering specifically has not been demonstrated in controlled human trials.

### 6.2. Ferroptosis and Redox Sensitivity

Ferroptosis, an iron-dependent, lipid-peroxidation-mediated form of programmed cell death, is described as a significant vulnerability of cancer cells under high oxidative stress [116,117]. Ferroptosis sensitivity is strongly influenced by cellular lipid composition, fatty acid metabolism, and antioxidant defense mechanisms. Polyunsaturated fatty acids (PUFAs) incorporated into membrane phospholipids, particularly through ACSL4- and LPCAT3-dependent pathways, promote susceptibility to lipid peroxidation, whereas monounsaturated fatty acids (MUFAs) may confer resistance. Altered lipid synthesis and remodeling can therefore influence tumor adaptation to oxidative stress and therapeutic resistance. In parallel, the cystine–glutathione–GPX4 axis and alternative antioxidant systems, including FSP1–CoQ10 and DHODH, protect cells against ferroptotic damage.

In this context, a mechanistic intersection between the H_2_O_2_ and free-radical burden produced by high-dose IV vitamin C and the induction of ferroptosis is biologically plausible; combination with targeting of the cystine/glutathione axis is a hypothesis for future research [118]. Macrophage-derived itaconate can be transported into tumor cells through SLC13A3, promoting resistance to ferroptosis and impairing antitumor immunity [119]. Ferroptosis induction is a promising target for future combination-therapy design, but no clinical data currently link high-dose vitamin C, or any other component discussed in this review, to ferroptosis-mediated tumor control in patients.

### 6.3. Autophagy/Amino-Acid Metabolism

Autophagy and amino-acid metabolism represent interconnected mechanisms through which tumor cells adapt to metabolic stress and the tumor microenvironment contributes to immune suppression [120]. Inhibition of lysosomal/autophagic flux, including with lysosomotropic agents such as hydroxychloroquine, has been investigated as a strategy to increase sensitivity to chemotherapy and targeted therapies, particularly in metabolically stressed and RAS-mutant tumors [121]. However, clinical studies have not demonstrated a consistent survival benefit, and autophagy inhibition remains investigational.

Amino-acid metabolic pathways also contribute to tumor-associated immune suppression. Increased arginase-1 activity and indoleamine 2,3-dioxygenase 1 (IDO1)-mediated tryptophan–kynurenine metabolism can impair T-cell function within the tumor microenvironment [111,112,118]. Therapeutic targeting of these pathways, including combinations with immune checkpoint inhibitors, has entered clinical investigation but has not yet established a standard therapeutic role [111]. Within amino acid metabolism, the targeting of glutaminolysis using glutamine analogs, DON prodrugs and specific glutaminase inhibitors has received increasing attention to inhibit tumor cell proliferation and enhance immune recognition [122,123,124,125]. Collectively, modulation of autophagy and amino-acid metabolism represents a biologically plausible strategy for overcoming metabolic adaptation and immune suppression, but its integration with other metabolically directed therapies requires further prospective clinical evaluation.

### 6.4. TME-Directed Strategies

The tumor microenvironment (TME), comprising cancer-associated fibroblasts (CAFs), tumor-associated macrophages (TAMs), abnormal vasculature, and immunosuppressive cytokine networks, represents an additional therapeutic layer beyond direct tumor cell targeting [126]. CAFs promote tumor progression and treatment resistance through extracellular matrix remodeling, growth factor and cytokine signaling, and interactions with immune cells; strategies targeting FAP-positive CAFs, CXCL12/CXCR4 signaling, and TGF-β are under clinical investigation [127,128]. Similarly, TAMs contribute to an immunosuppressive microenvironment and resistance to systemic therapy, although their functional heterogeneity has shifted therapeutic interest from broad macrophage depletion toward selective reprogramming approaches [2,129,130].

Abnormal tumor vasculature further contributes to hypoxia and immune exclusion [131]. Vascular-normalization strategies, particularly those involving VEGF-pathway inhibition, may improve perfusion and immune-cell infiltration and provide a rationale for combining anti-angiogenic agents with immune checkpoint inhibitors in selected cancers [132]. In parallel, cytokine pathways such as IL-6/JAK/STAT3 and TGF-β mediate bidirectional signaling among tumor, stromal, and immune cells and are being investigated as therapeutic targets, although their effects are highly context dependent [133,134].

Collectively, CAF-, TAM-, vascular-, and cytokine-directed strategies illustrate the therapeutic potential of remodeling the TME rather than targeting cancer cells alone [135]. However, most of these approaches remain preclinical or in early clinical development, and their integration with metabolically directed interventions, including hyperthermia, has not been prospectively validated [136]. They should therefore be considered emerging targets for mechanistic and clinical investigation rather than established components of a metabolically directed treatment regimen.

## 7. Clinical Evidence Hierarchy and Translational Status

To distinguish established therapies from investigational and experimental approaches, interventions are grouped into three evidence categories. The first category includes clinically validated therapies, such as targeted therapies, monoclonal antibodies, immune checkpoint inhibitors, and selected regional hyperthermia applications. The second category comprises clinically investigated metabolic interventions, including fasting-mimicking diets, ketogenic diets, high-dose intravenous vitamin C, and metabolically supported chemotherapy, each supported by varying levels of clinical evidence. The third category includes experimental or unproven strategies, such as emerging metabolic targets, investigational drug-delivery platforms, DMSO-mediated chemotherapy delivery, and historical low-dose insulin potentiation therapy. These classifications reflect the evidence supporting individual interventions rather than the efficacy of combined metabolic regimens. Table 1 summarizes their clinical evidence and limitations.

Evidence categories reflect the clinical maturity of each intervention for its specified indication. Regulatory approval of a delivery platform or formulation does not imply clinical validation of its application to metabolic cancer therapy. Similarly, evidence from randomized trials investigating one dietary intervention should not be extrapolated to other dietary strategies.

## 8. Translational Roadmap for Prospective Trials

The biological rationale for integrating metabolically directed interventions with standard oncologic treatment warrants prospective investigation; however, current evidence does not support a predefined multicomponent treatment sequence. Future studies should therefore evaluate individual interventions and biologically justified combinations within formally approved prospective protocols. Early-phase studies should first establish feasibility, safety, and dose compatibility when these interventions are added to guideline-based anticancer therapy. Whenever possible, study designs should allow the contribution of individual components to be distinguished, particularly when interventions supported by different levels of clinical evidence are combined. Subsequent randomized or appropriately controlled trials should determine whether these approaches provide measurable benefit beyond standard therapy. Simultaneous introduction of multiple investigational components should generally be avoided unless supported by adequate preclinical and safety data, as this complicates attribution of efficacy and toxicity.

Patient selection should consider tumor type, disease stage, molecular characteristics, prior treatment, nutritional status, and relevant metabolic or microenvironmental features. Correlative biomarkers of metabolic activity, hypoxia, immune status, and treatment-induced metabolic adaptation should be incorporated where appropriate. Experimental approaches such as extracellular flux analysis may support biological characterization and hypothesis generation but should not currently guide clinical treatment selection. Clinical endpoints should reflect the phase and objective of the study: early trials should prioritize safety, tolerability, feasibility, and dose-limiting toxicity, whereas subsequent studies may assess objective response, progression-free survival, overall survival, quality of life, and treatment-related toxicity. Pharmacodynamic and biomarker endpoints may additionally help determine whether the intended metabolic or microenvironmental effect is achieved in vivo.

Safety monitoring should be intervention specific. High-dose intravenous vitamin C requires renal assessment and consideration of G6PD deficiency; insulin-based approaches require intensive glucose monitoring and predefined hypoglycemia management; nutritional interventions require assessment of nutritional status and the risk of weight or lean-mass loss; and HBOT and hyperthermia require their respective eligibility and safety assessments. Monitoring procedures, stopping rules, and adverse-event reporting should be prespecified. All prospective studies should be conducted under institutional ethics/IRB approval, with multidisciplinary oncologic oversight and informed consent clearly communicating the evidence level and investigational status of each component. Metabolically directed interventions should be evaluated alongside, rather than presented as established alternatives to guideline-based oncologic care. Future clinical development should prioritize biomarker-informed studies that distinguish mechanistic plausibility from clinically meaningful therapeutic benefit.

## 9. Limitations and Scientific Caveats

Several limitations should be considered when interpreting the approaches discussed in this review. First, the evidence base is highly heterogeneous, ranging from mechanistic and preclinical studies to case reports, retrospective series, early-phase trials, and randomized clinical studies. These different levels of evidence are not directly comparable, and findings obtained in specific tumor types or experimental models should not be generalized across cancers. Importantly, no multicomponent regimen integrating several of the metabolic interventions reviewed here has been validated as a whole in a randomized controlled trial. The contribution of each component therefore needs to be evaluated independently before drawing conclusions regarding combined efficacy. Second, several proposed interactions between interventions remain mechanistic hypotheses rather than clinically demonstrated synergies. These include the proposed use of DMSO beyond its established role as a pharmaceutical excipient, potentiation of anticancer effects through combinations of HBOT with high-dose vitamin C or other metabolic interventions, and assumptions about insulin-mediated enhancement of chemotherapy outside specifically studied protocols. Similarly, the available literature does not support DMSO as an anticancer agent or historical low-dose insulin potentiation therapy (IPT) as an established method of enhancing chemotherapy. Because hyperbaric oxygen as a standalone treatment has been reported to produce both anti-tumoral and tumor-favoring effects in some tumor types, HBOT indication should be individualized by tumor type in light of current literature, and no synergistic efficacy with other treatment components in this review has been clinically established. Much of the evidence supporting complex metabolically directed combinations comes from small, uncontrolled, or single-center studies. These observations are valuable for hypothesis generation but cannot establish causality, determine the contribution of individual treatment components, or substitute for controlled clinical trials. Accordingly, favorable outcomes reported in individual patients or small cohorts should not be interpreted as evidence of efficacy for the combined regimen. These limitations reinforce the need for stepwise, tumor-specific, biomarker-informed prospective studies that evaluate safety and efficacy against clearly defined standard-of-care comparators.

## 10. Conclusions

Deliberately targeting tumor metabolism alongside standard oncological care is a scientifically coherent and active research direction, but the individual components discussed in this review sit at markedly different points on the evidence spectrum. Genetically targeted therapy, monoclonal antibodies, immune checkpoint inhibition, and—among metabolic interventions—a structured fasting-mimicking diet and hyperthermia in selected tumor types are supported by randomized or phase III evidence. High-dose intravenous vitamin C, hyperbaric oxygen therapy, and a specific monitored protocol combining standard-dose chemotherapy with induced hypoglycemia remain investigational, with case-report to phase I/II-level support, as well as a challenging funding environment due to off-patent or non-patentable nature. DMSO as an anticancer carrier and historic low-dose Insulin Potentiation Therapy are, on current evidence, unproven and should not be presented to patients as effective cancer treatments. Nanoparticle, exosomal, and peptide-targeted drug-delivery platforms offer a considerably better-evidenced alternative direction for improving tumor-selective drug delivery than non-targeted solvent carriers.

We envision combinatory diet-drug multimodal treatments as a promising research direction in the emerging field of metabolic oncology and encourage more scientific interest and public funding to quantify the weight of each component. Although combining metabolically directed interventions may have a mechanistic rationale, additive or synergistic clinical benefits cannot be assumed without prospective validation. Future investigations should therefore evaluate individual metabolic interventions and biologically justified combinations to further substantiate and expand their levels of clinical evidence.

## Figures and Tables

**Table 1:** Evidence-based classification and clinical maturity of metabolic interventions, targeted therapies, and drug-delivery strategies in oncology.

Component	Mechanistic Hypothesis	Evidence Category	Level of Clinical Evidence	Note
Targeted smart drugs (EGFR/ALK/BRAF/PARP inhibitors)	Driver-mutation-specific inhibition	Established	Strong (Phase III, standard treatment)	Evidence-based oncology; used within its indication regardless of the rest of this review.
Monoclonal antibodies (trastuzumab, bevacizumab)	Receptor blockade/anti-angiogenesis	Established	Strong (Phase III, standard treatment)	Proven standard treatment in biomarker-selected patients.
Immune checkpoint inhibitors (PD-1/PD-L1)	Removal of T-cell suppression	Established	Strong (Phase III, standard treatment)	Indicated according to PD-L1/MSI/TMB; already evidence-based.
Fasting-mimicking diet (FMD)	Differential stress resistance; modulation of glucose and IGF-1 signaling	Clinically Investigated	Moderate (randomized Phase II)	Randomized evidence exists, but survival benefit is unproven; toxicity outcomes are inconsistent.
Ketogenic diet (KD)	Nutritional ketosis; modulation of glucose availability and metabolic signaling	Clinically Investigated	Limited (small, heterogeneous clinical studies)	Feasibility and metabolic effects investigated; insufficient evidence of improved survival or antitumor efficacy in monotherapy.
Metronomic chemotherapy	Anti-angiogenesis, immune modulation	Established in selected settings	Moderate (Phase II/III in some tumors, mostly palliative)	Overall survival benefit not demonstrated in most studies, influenced by the recurrent/palliative settings.
Regional/systemic hyperthermia	Protein denaturation, increased blood flow, immunogenic cell death	Established in selected indications	Moderate–Strong (Phase III in some tumor types, as an adjunct)	Evidence varies by tumor type; proven as part of a combination, not alone.
High-dose IV vitamin C	Peroxide-mediated pro-oxidant cytotoxicity	Clinically Investigated	Limited (Phase I/II, safety-focused; no Phase III survival data)	Benefit shown in quality of life/tolerance; insufficient evidence of stand-alone anti-tumor efficacy.
Hyperbaric oxygen therapy	Reduction of tumor hypoxia/HIF-1α	Investigational	Limited and conflicting (mixed preclinical data)	Both anti-tumoral and proliferation-favoring effects reported in some cancer types; individualize by tumor type.
Metabolically supported chemotherapy (İyikesici/Seyfried protocol): standard-dose chemotherapy + induced hypoglycemia + KD/HT/HBOT	Hypothesized insulin-mediated metabolic modulation and chemotherapy sensitization	Clinically Investigated	Limited (case reports and retrospective case series; no randomized trials)	Distinct from IPT: retains standard chemotherapy dose; requires continuous glucose monitoring; not independently replicated.
DMSO infusion (as chemotherapy carrier/anticancer agent)	Increased cell-membrane permeability	Unproven	Unproven for anticancer effect (no human efficacy data)	Reviews state there is no evidence it treats cancer in humans; limited signal for pain palliation only.
Insulin Potentiation Therapy (IPT), historic low-dose concept	Assumption that insulin increases intracellular drug uptake at 10–25% chemotherapy dose	Unproven	Unproven/not recommended by expert organizations	NCI, MSKCC, and CAM-Cancer describe IPT as unproven and potentially harmful (hypoglycemia, resistance risk from inadequate dosing).
Nanoparticle-based drug delivery	Controlled release and improved delivery of therapeutic agents, including potential metabolic inhibitors	Established for selected formulations; investigational for metabolic targeting	Mixed (approved oncology formulations; metabolic applications largely preclinical–early clinical)	Approved nanomedicines exist, but their approval does not establish efficacy for metabolic cancer targeting.
Exosome-based drug delivery	Vesicle-mediated transport of therapeutic cargo, including metabolic regulatory molecules	Investigational	Limited (predominantly preclinical; early clinical development)	No established clinical efficacy for exosome-mediated metabolic cancer therapy.
Peptide-based targeted delivery	Receptor-mediated targeting and delivery of therapeutic agents	Investigational (preclinical to early clinical)	Variable (platform-dependent; metabolic applications largely experimental)	Some peptide-targeted oncology therapies are clinically established, but this does not validate investigational peptide carriers for metabolic inhibitors.
Tumor-microenvironment targeting (CAF/TAM/vascular normalization/cytokine network)	Reprogramming of the stromal-immune support system	Investigational (preclinical to early clinical)	Research stage (preclinical–early clinical; some combinations approved, most experimental)	Bevacizumab+atezolizumab combination approved; CSF1R- and IL-6-targeted approaches have shown mixed results in early-phase trials.
Combined multi-component ‘metabolically controlled’ protocol as a whole (all elements above given together)	Simultaneous multi-target metabolic and immunological suppression	Unvalidated combination	Not tested as a combined regimen (no trial of the full combination exists)	Each component must be evaluated on its own evidence level; the combination itself requires a prospective, controlled trial before any efficacy claim can be made.

## Data Availability

No new data were created, all data discussed are available in the cited references.
